# Co-deficiency of B7-H3 and B7-H4 identifies high CD8 + T cell infiltration and better prognosis in pancreatic cancer

**DOI:** 10.1186/s12885-022-09294-w

**Published:** 2022-02-26

**Authors:** Shuping Si, Lei Wang, Hui Cao, Yuhua Xu, Qiang Zhan

**Affiliations:** Department of Gastroenterology, Wuxi People’s Hospital Affiliated to Nanjing Medical University, No. 299 Qing Yang Road, Wuxi, 214023 China

**Keywords:** B7-H3, B7-H4, prognosis, Immune cell infiltration, pancreatic cancer

## Abstract

**Background:**

Immunotherapy is a novel hotspot for the treatment of pancreatic adenocarcinoma (PAAD). However, potential biomarkers which could identify the inflamed tumor microenvironment (TME) are urgently required.

**Methods:**

In the present study, we measured the levels of B7-H3, B7-H4, and major tumor-infiltrating immune cells (TIICs) using bioinformatics analyses and immunohistochemistry (IHC) staining on PAAD samples represented in the tissue microarray (TMA) format. Statistical analysis and figures exhibition were performed using R 4.1.0, SPSS 26.0, and GraphPad Prism 6.0.

**Results:**

B7-H3 and B7-H4 were up-regulated in PAAD compared with para-tumor tissues, and their expression exhibited no tight correlation in PAAD tissues. B7-H3 and B7-H4 were lowly expressed in well-differentiated PAAD tissues and correlated with poorly differentiated grades. Besides, single B7-H3 or B7-H4 expression exhibited limited prognostic value, but co-deficiency of B7-H3 and B7-H4 predicted a better prognosis in PAAD. Moreover, co-deficiency of B7-H3 and B7-H4 indicated immuno-hot tumors with high CD8 + T cell infiltration.

**Conclusions:**

Overall, combined B7-H3 and B7-H4 expression is a promising stratification strategy to assess prognosis and immunogenicity in PAAD, which could be used as a novel classifier in clinical practice.

**Supplementary Information:**

The online version contains supplementary material available at 10.1186/s12885-022-09294-w.

## Background

Pancreatic adenocarcinoma (PAAD) is one of the most fatal malignant tumors in the world, featured with dreadful invasiveness, powerful proliferative potential, and poor clinical outcome. The early diagnosis of PAAD is rare on account of the obscure symptoms, and the morbidity of PAAD has been significantly elevated over the last few decades. Although PAAD does not account for a high proportion of all patients, survival is lowest for cancers of the pancreas (10%) [1]. With the rapid progress of emerging therapeutic programs, immunotherapy is becoming a promising hotspot for the treatment of PAAD [2]. It has been revealed that the response for immunotherapy is low in PAAD due to its non-inflamed tumor microenvironment (TME) [3–5]. Growing evidence indicates that tumor progression and therapeutic response were critically affected by host immune response, which depends on the abundance of tumor-infiltrating immune cells (TIICs) in TME [6, 7]. Thus, potential biomarkers which could identify the abundance of TIICs in TME of PAAD are urgently required in clinical practice.

In recent years, the roles of co-stimulatory B7 family molecules in regulating tumor immunity have been widely concerned, specially programmed cell death ligand 1 (PD-L1), also named as B7-H1 [8]. PD-L1 expression is usually correlated with inflamed TME phenotype and predicts a high response rate to immunotherapy in the clinic [9, 10]. In addition to B7-H1, B7-H3 and B7-H4 are becoming promising hotspots [11]. According to previous reports, B7-H3 and B7-H4 are significantly up-regulated in PAAD tissues compared with non-tumor or normal pancreas tissues [12, 13]. Besides, co-expressed or mutually-exclusive patterns of B7 molecules predict inflamed or non-inflamed TME in multiple human cancers [14, 15]. However, the correlation between B7-H3 and B7-H4 expression and TIICs abundance as well as the predictive value of combined B7-H3 and B7-H4 in assessing prognosis has not been investigated yet.

In this research, we first analyzed the expression of B7-H3 and B7-H4 as well as their associations between clinic-pathological features in PAAD. Besides, the prognostic values and immuno-correlations of B7-H3, B7-H4, and combined expression were also evaluated. As result, we found that B7-H3 and B7-H4 were upregulated in PAAD tissues and correlated with advanced differentiated grades. Moreover, co-deficiency of B7-H3 and B7-H4 in PAAD predicted better clinical outcomes and identifies high CD8 + T cell infiltration. Overall, co-deficiency of B7-H3 and B7-H4 is a promising prognostic and immunogenic biomarker in PAAD.

## Methods

### Acquisition of TCGA data

Normalized RNA-sequencing (RNA-seq) data and corresponding clinical information of PAAD samples in the Cancer Genome Atlas (TCGA) database were downloaded from the UCSC Xena website (https://xenabrowser.net/datapages/). Patients with missing or insufficient data were excluded from this research. Finally, a total of 178 tumor samples were retained for further analysis.

### Analyses of the GEPIA and CPTAC databases

GEPIA (http://gepia.cancer-pku.cn/) was an interactive website based on the TCGA and GTEx databases and used for RNA expression analyses [16]. In the present study, the GEPIA website was used to explore the expression levels of B7-H3 and B7-H4 in PAAD and adjacent pancreas tissues. In addition, to further compare the differential expressions of B7-H3 and B7-H4 at protein levels, the proteome data of the CPTAC dataset (http://ualcan.path.uab.edu/analysis-prot.html) were also used for differential analyses of B7-H3 and B7-H4 [17].

### Immune infiltration analysis

Tumor Immune Estimation Resource (TIMER) database is an online tool for systematic analysis of immune cell infiltration across diverse cancer types from TCGA [18]. We evaluated the correlation of B7-H3 & B7-H4 expressions with the infiltration of main types of immune cells, including B cells, CD8 + T cells, CD4 + T cells, neutrophils, macrophages, and dendritic cells (DCs).

The relative abundance of more types of infiltrating immune cells was analyzed using the xCell algorithm (https://xcell.ucsf.edu/), an emerging tool to estimate the abundance of 64 immune and stromal cell types based on gene expression profiles [19]. Pre-calculated infiltrating data of TIICs corresponding to TCGA-PAAD samples were downloaded from the xCell website.

### Clinical samples

The PAAD tissue microarray (TMA, Cat. no HPanA150CS04) was purchased from Outdo BioTech (Shanghai, China). A total of 120 PAAD and 30 paired para-tumor tissues were included in the TMA. Detailed clinic-pathological characteristics of these patients were also provided by Outdo BioTech. Ethical approval for the use of the TMA was granted by the Clinical Research Ethics Committee (Outdo BioTech).

### Immunohistochemistry

Immunohistochemistry (IHC) staining was performed on the TMA of PAAD tissues. The primary antibodies used in the research were as follows: anti-B7-H3 (1:8000 dilution, Cat. no ab219648, Abcam, Cambridge, UK), anti-B7-H4 (1:50 dilution, Cat. no ab252438, Abcam, Cambridge, UK), and anti-CD8 (Ready-to-use, Cat. no PA067, Abcarta, Suzhou, China). Antibody staining was visualized using diaminobenzidine (DAB) and hematoxylin counterstain, and stained TMA was scanned using Aperio Digital Pathology Slide Scanners.

### Semi-quantitative assessment

A total of 104 TMA points were retained for further analysis after the exfoliated points were removed. All stained points were independently assessed by two senior pathologists. For semi-quantitative evaluation of B7-H3 and B7-H4 staining, the percentage of positively stained tumor cells was scored as 0–4: 0 (< 1%), 1 (1–5%), 2 (6–25%), 3 (26–50%) and 4 (> 50%). The staining intensity was scored as 0–3: 0 (negative), 1 (weak), 2 (moderate) and 3 (strong). The immune-reactivity score (IRS) equals the percentages of positive cells multiplied with staining intensity. For semi-quantitative evaluation of CD8 staining, the infiltration level of CD8 + immune cells was evaluated by estimating the percentage of cells with strong intensity of membrane staining in the stroma cells [20].

### Statistical analysis

Statistical analysis and figures exhibition were performed using R 4.1.0, SPSS 26.0, and GraphPad Prism 6.0. Most of the data were analyzed by Student’s t-test, Mann–Whitney test, and one-way ANOVA. Kaplan–Meier survival plots were generated with survival curves compared by log-rank test. The Chi-square test was used to assess differences in clinic-pathological features between groups with different risks. For all analyses, differences were deemed statistically significant when P-value was less than or equal 0.05.

## Results

### B7-H3 and B7-H4 are up-regulated in PAAD compared with para-tumor tissues

**Fig. 1:**
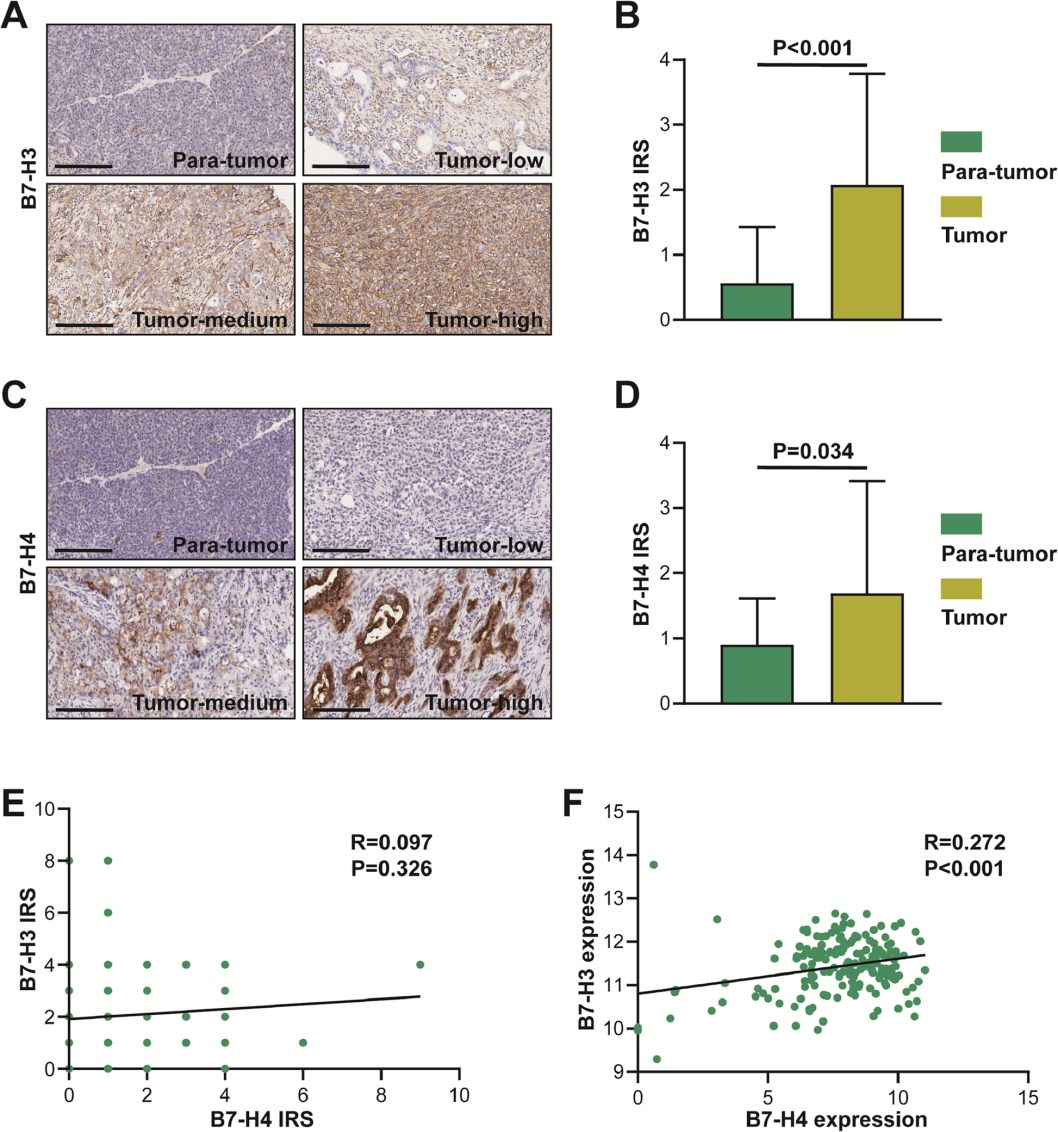
Expression levels of B7-H3 and B7-H4 PAAD tissues**. A** Representative microphotographs revealing low B7-H3 expression in para-tumor tissues and low, medium, and high B7-H3 expression in tumor tissues using IHC staining. Brown, B7-H3. Blue, haematoxylin. Bar = 200 μm. B7-H3 was mostly localized to the cytomembrane of tumor cells and tumor stroma. **B** The semi-quantitative analysis of B7-H3 in tumor and para-tumor tissues. B7-H3 was significantly up-regulated in tumor tissues compared with para-tumor tissues. **C** Representative microphotographs revealing low B7-H4 expression in para-tumor tissues and low, medium, and high B7-H4 expression in tumor tissues using IHC staining. Brown, B7-H4. Blue, haematoxylin. Bar = 200 μm. B7-H4 was mostly localized to the cytomembrane of tumor cells but not tumor stroma **D** The semi-quantitative analysis of B7-H4 in tumor and para-tumor tissues. B7-H4 was significantly up-regulated in tumor tissues compared with para-tumor tissues. **E** Correlation between B7-H3 and B7-H4 expression in the TMA cohort. No obvious correlation was found between B7-H3 and B7-H4 expression. **F** Correlation between *B7-H3* and *B7-H4* mRNA expression in the TCGA database. B7-H3 was positively correlated with B7-H4 expression

As described previously, several research groups reported that B7-H3 and B7-H4 are up-regulated in multiple cancers [21, 22]. In the GEPIA and CPTAC databases, B7-H3 was upregulated in PAAD tissues, while B7-H4 showed no difference between tumor and para-tumor tissues (Figure S1A-D). We also assessed B7-H3 and B7-H4 expression based on IHC staining. As shown in Fig. 1A, the immuno-reactivity of B7-H3 was mostly localized to the cytomembrane of tumor cells and tumor stroma. After the semi-quantitative analysis, we found that the IRS of B7-H3 in PAAD tissues was significantly higher than para-cancerous tissues (Fig. 1B). Similar to B7-H3, the immuno-reactivity of B7-H4 was also localized to the cytomembrane of tumor cells and but not stroma (Fig. 1C). Besides, the expression of B7-H4 was notably up-regulated in PAAD tissues compared with para-cancerous tissues (Fig. 1D). We also evaluated the correlation between B7-H3 and B7-H4 expression, and the results showed that the protein expression of B7-H3 and B7-H4 had no obvious correlation (Fig. 1E). However, in the TCGA database, B7-H3 mRNA was positively correlated with B7-H4 mRNA (Fig. 1F). Overall, these data suggest that the expression of B7-H3 and B7-H4 proteins are up-regulated in PAAD tissues and have no notable correlation.

### B7-H3 and B7-H4 are lowly expressed in well-differentiated PAAD tissues

**Fig. 2:**
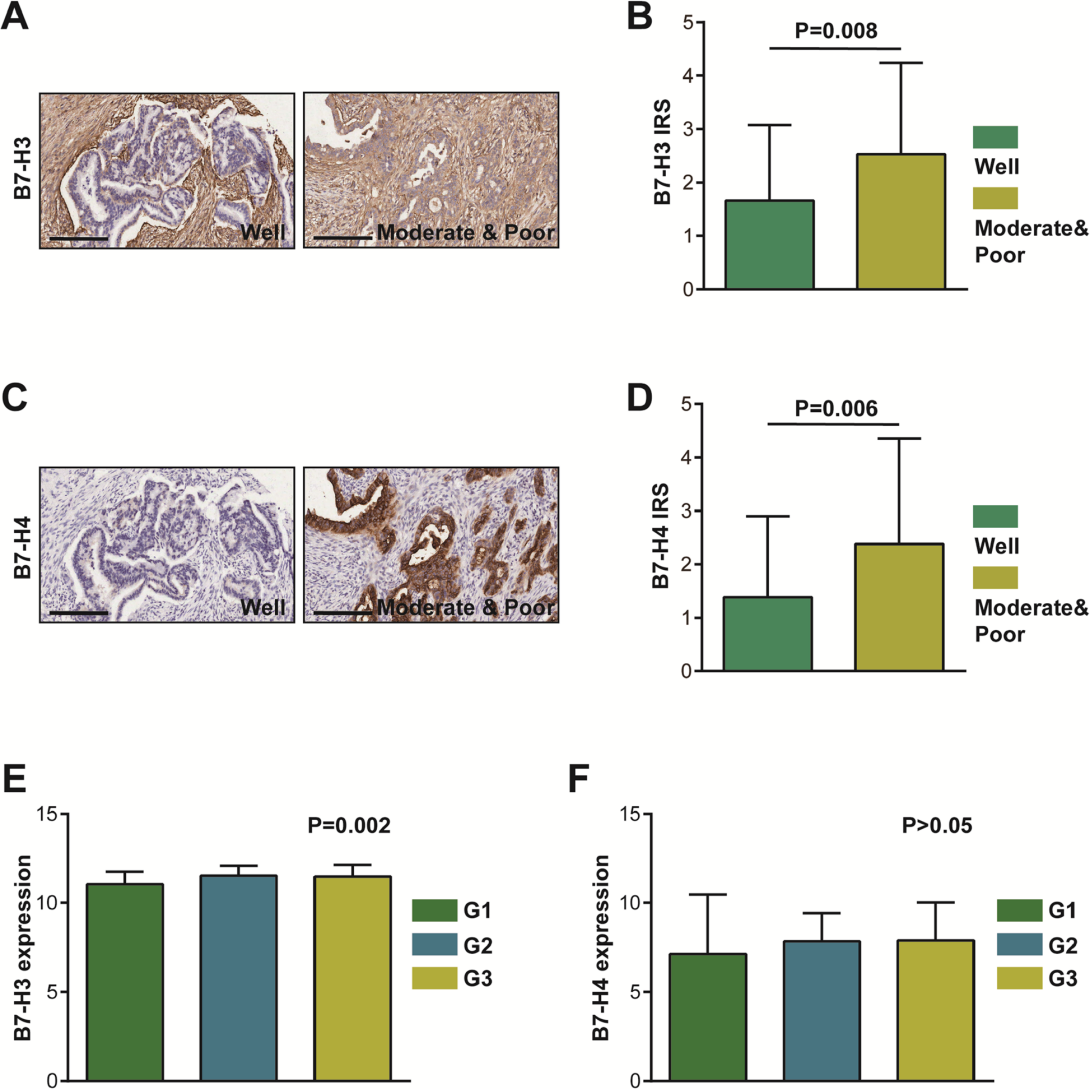
Expression levels of B7-H3 and B7-H4 in variously differentiated PAAD tissues. **A** Representative microphotographs revealing low B7-H3 expression in well-differentiated tissues and high B7-H3 expression in moderate and poor-differentiated tissues using IHC staining. Brown, B7-H3. Blue, haematoxylin. Bar = 200 μm. **B** The semi-quantitative analysis of B7-H3 in variously differentiated PAAD tissues. B7-H3 was significantly up-regulated in moderate and poor-differentiated tissues compared with well-differentiated tissues. **C** Representative microphotographs revealing low B7-H4 expression in well-differentiated tissues and high B7-H4 expression in moderate and poor-differentiated tissues using IHC staining. Brown, B7-H4. Blue, haematoxylin. Bar = 200 μm. **D** The semi-quantitative of B7-H4 in variously differentiated PAAD tissues. B7-H4 was significantly up-regulated in moderate and poor-differentiated tissues compared with well-differentiated tissues. **E** *B7-H3* mRNA expression was various in differently differentiated PAAD tissues in the TCGA database. **F** *B7-H4* mRNA expression showed no changes in differently differentiated PAAD tissues in the TCGA database

Next, the associations between clinic-pathological features and B7 molecules expression were evaluated in the current patients’ cohort. As shown in Table 1, the expression levels of B7-H3 and B7-H4 were not associated with gender, age, T stage, N stage, M stage, and clinical stage. However, these two B7 molecules were significantly associated with differentiation (Table 1). We next compared the expression levels of B7-H3 and B7-H4 in well-differentiated and moderate & poor-differentiated groups, and the results exhibited that B7-H3 and B7-H4 were notably downregulated in well-differentiated PAAD tissues (Fig. 2A-D). Besides, in the TCGA database, B7-H3 was significantly correlated with advanced differentiated grades (Fig. 2E). Although B7-H4 tended to be upregulated with advanced differentiated grades, the difference was not statistically significant (Fig. 2F). Overall, deficiency of B7-H3 and/or B7-H4 identifies well-differentiated tumors in PAAD.

**Table 1:** Association between B7-H3 & B7-H4 expression and clinic-pathological parameters in PAAD

Clinic-pathological parameters	Cases	B7-H3 expression		χ^2^ value	*P* value	B7-H4 expression		χ^2^ value	*P* value
		Low	High			Low	High		
Gender									
Female	46	24	22	0.156	0.693	24	22	0.698	0.404
Male	58	28	30			35	23		
Age									
≤ 60	47	25	22	0.349	0.554	22	25	3.439	0.064
> 60	57	27	30			37	20		
Differentiation									
Well	65	39	26	5.455	0.020	42	23	6.292	0.012
Moderate & poor	34	12	22			13	21		
T stage									
T1-2	29	17	12	0.829	0.363	19	10	1.466	0.226
T3-4	56	27	29			29	27		
N stage									
N0	48	25	23	0.157	0.692	28	20	0.009	0.925
N1	54	26	28			31	23		
M stage									
M0	98	51	47	/	0.205*	58	40	/	0.083*
M1	6	1	5			1	5		
Clinical stage									
1-2A	46	25	21	0.624	0.430	27	19	0.130	0.719
2B-4	58	27	31			32	26		

Note: *P value was calculated by Fisher test

### Correlations between B7-H3 & B7-H4 and infiltration of main types of immune cells

**Fig. 3:**
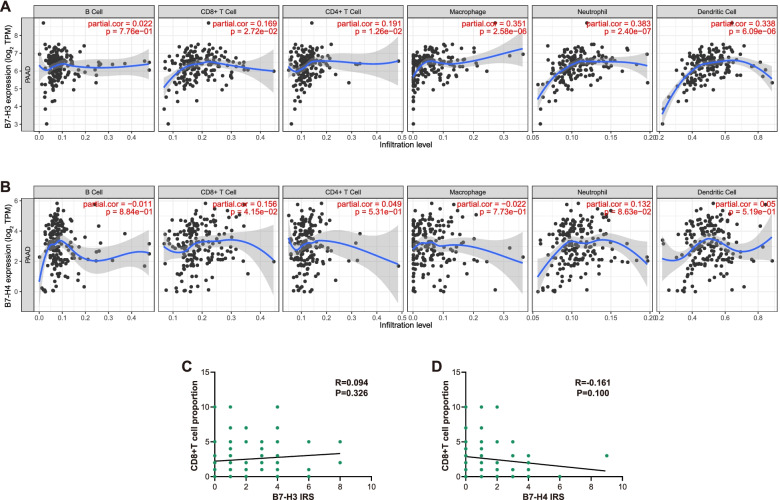
Correlations between B7-H3 & B7-H4 and infiltration of main types of immune cells. **A**, **B** Correlations between B7-H3 & B7-H4 expression levels and the infiltration of six immune cells. B7-H3 was positively correlated with CD8 + T cells, CD4 + T cells, neutrophils, macrophages, and DCs, while B7-H4 was only positively correlated with CD8 + T cells. **C**, **D** Correlations between B7-H3 & B7-H4 expression levels and the infiltration of CD8 + T cell. Neither B7-H3 nor B7-H4 was correlated with CD8 + T cell infiltration

Given B7-H3 & B7-H4 were correlated with TIICs in other cancers [23, 24], we also assessed the correlations between B7-H3 & B7-H4 and infiltration of main types of immune cells. B7-H3 was positively correlated with CD8 + T cells, CD4 + T cells, neutrophils, macrophages, and DCs, while B7-H4 was only positively correlated with CD8 + T cells (Fig. 3A-B). To validate the results, we performed IHC staining using anti-CD8 antibody. However, neither B7-H3 nor B7-H4 was correlated with CD8 + T cell infiltration (Fig. 3C-D). Thus, the correlations between B7-H3 & B7-H4 and immune cells infiltration are contradictory and need to be further confirmed.

### Co-deficiency of B7-H3 and B7-H4 predicts a better prognosis

**Fig. 4:**
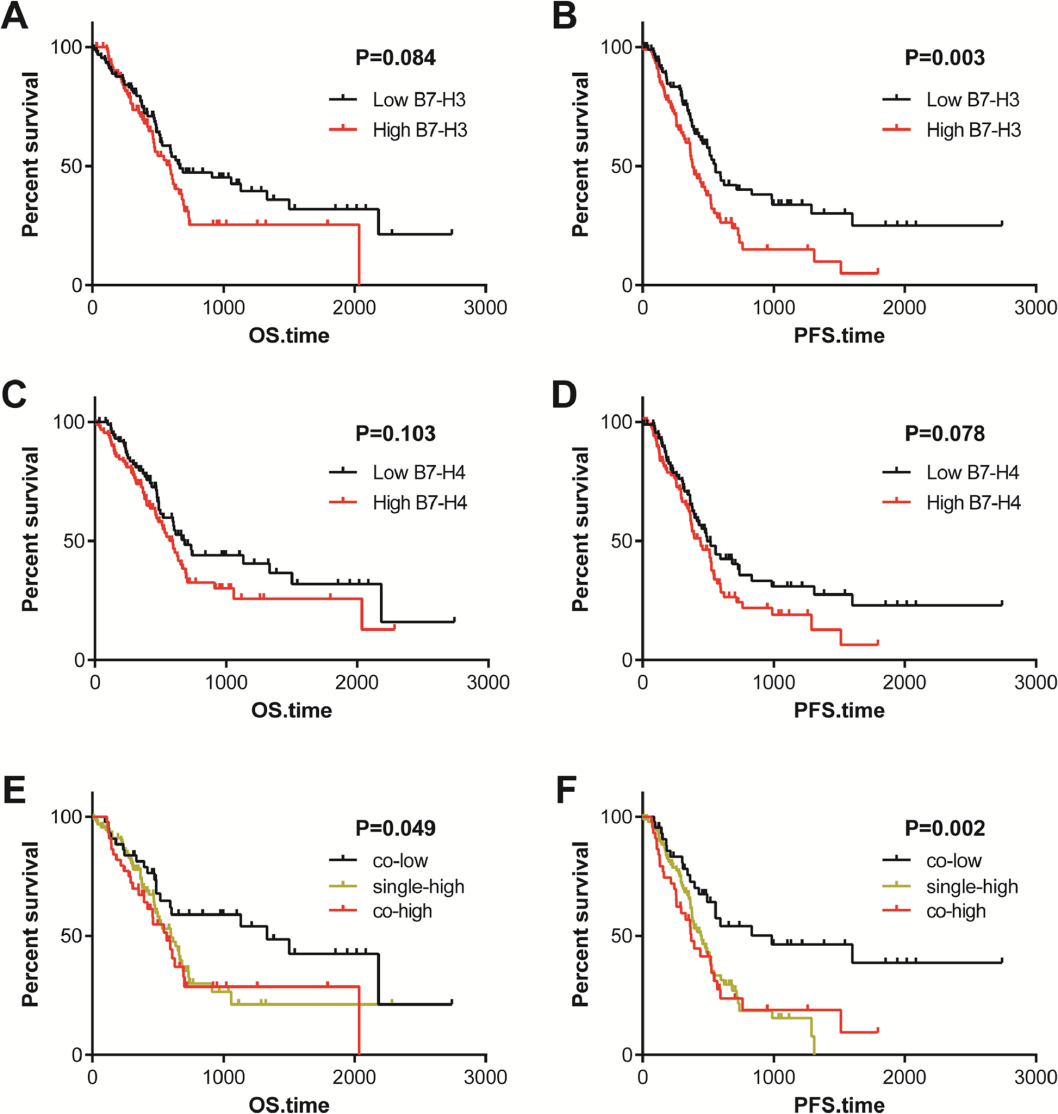
Survival plots of B7-H3 & B7-H4 in PAAD patients. **A**, **B** Prognostic value of B7-H3 expression in PAAD patients in term of OS and PFS. High B7-H3 expression was associated with a poor PFS but not associated with OS. **C**, **D** Prognostic value of B7-H4 expression in PAAD patients in term of OS and PFS. B7-H4 expression was not associated with OS and PFS. **E**, F Prognostic value of combined B7-H3 & B7-H4 expression in PAAD patients in term of OS and PFS. Co-deficiency of B7-H3 and B7-H4 was associated with a better prognosis

We further definite the prognostic values of these two B7 molecules in patients with PAAD. Patients in the TCGA cohort were divided into low (*n* = 89) and high (*n* = 89) groups at the cut-off value of the median expression. The Kaplan–Meier curves exhibited B7-H3 and B7-H4 could not effectively predict overall survival (OS) in patients with PAAD (Fig. 4A, C). In term of progression-free survival (PFS), patients with high B7-H3 expression had a significantly worse prognosis than those with low expression (Fig. 4B). However, B7-H4 could not effectively predict PFS in PAAD patients (Fig. 4D). Furthermore, combined B7-H3 and B7-H4 expression was a promising prognostic biomarker. Co-deficiency of B7-H3 and B7-H4 predicted better prognosis in terms of both OS and PFS (Fig. 4E-F) in PAAD. Taken together, these results indicated that co-deficiency of B7-H3 and B7-H4 was a favorable prognostic factor in PAAD patients.

### Co-deficiency of B7-H3 and B7-H4 indicates high CD8 + T cell infiltration

**Fig. 5:**
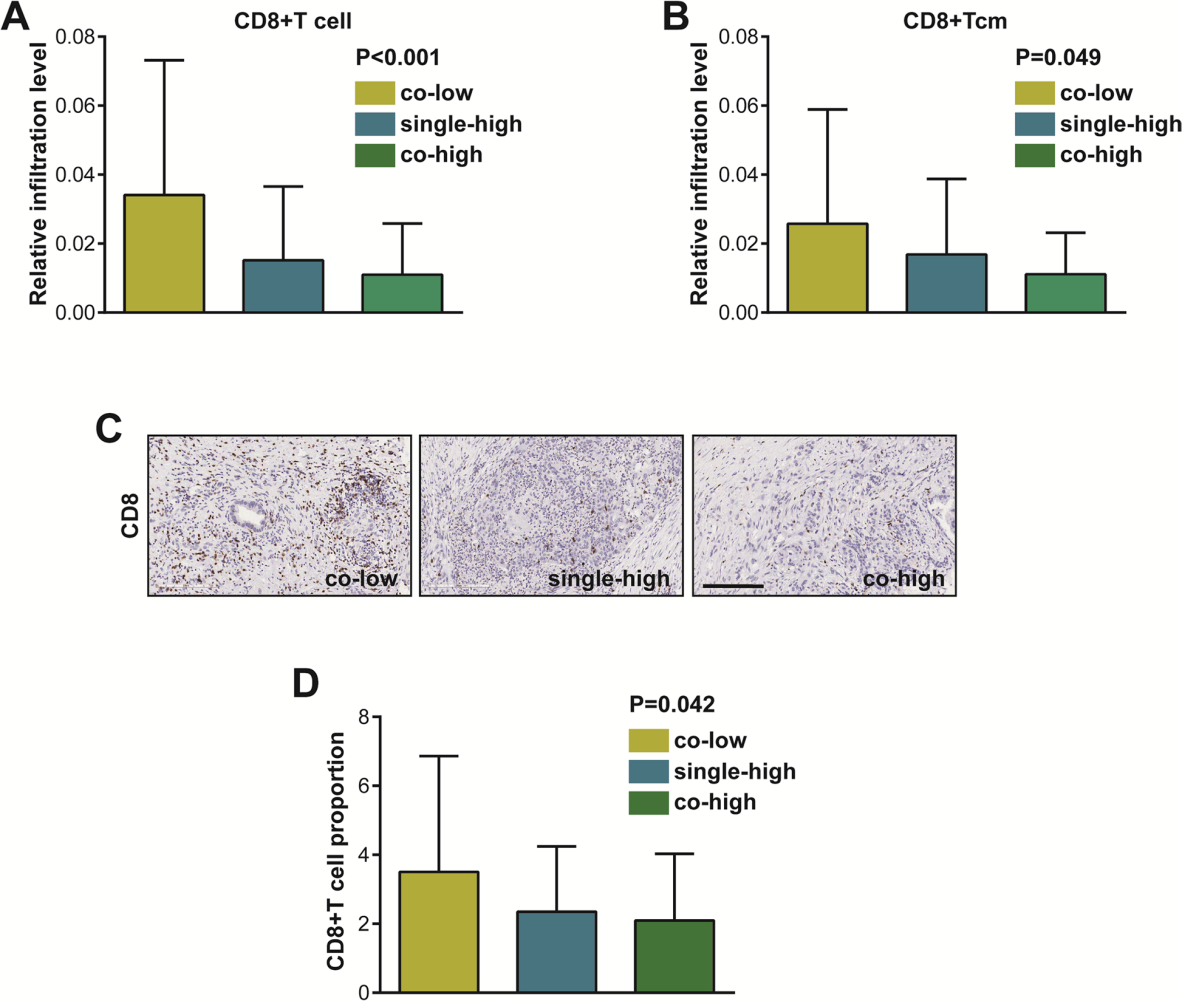
Various infiltration of CD8 + T cell in co-low, single-high and co-high groups. **A** The infiltration of total CD8 + T cell estimated by xCell algorithm was various in co-low, single-high, and co-high groups. **B** The infiltration of total CD8 + Tcm cell estimated by xCell algorithm was various in co-low, single-high, and co-high groups. **C** Representative microphotographs revealing various infiltration of CD8 + T cells using IHC staining. Brown, CD8. Blue, haematoxylin. Bar = 200 μm. **D** The infiltration of CD8 + T cell estimated by IHC staining was various in co-low, single-high and co-high groups

Given co-expressed or mutually-exclusive patterns of B7 molecules predict inflamed or non-inflamed TME in multiple human cancers [14, 15], we next assess whether co-deficiency of B7-H3 and B7-H4 predicted specific TME features. The xCell tool was used to estimate the abundance of 64 immune and stromal cell types in the TCGA database, and the abundance of these cells in the co-low, single-high and co-high groups were next compared. A subset of non-tumor cells was different in the three groups, and total CD8 + T cells and CD8 + Tcm cells were increased in the co-low groups (Table 2, Fig. 5A-B). As expected, the infiltrating abundance of CD8 + T cell was highest in the co-low group among these three groups (Fig. 5C-D). Overall, co-deficiency of B7-H3 and B7-H4 predicts high CD8 + T cell infiltration, which may explain the better prognosis in the co-low group of PAAD patients.

**Table 2:** Differences of immune cells levels estimated by xCell algorithm

Immune cells	Average			*F* value	*P* value
	**co-low**	**single-high**	**co-high**		
aDC	0.080	0.108	0.097	2.582	0.079
Adipocytes	0.108	0.076	0.031	3.456	0.034
Astrocytes	0.040	0.087	0.106	13.338	0.000
B cells	0.108	0.111	0.062	0.538	0.585
Basophils	0.038	0.012	0.007	6.142	0.003
CD4 + memory T cells	0.137	0.125	0.107	1.028	0.360
CD4 + naïve T cells	0.060	0.064	0.045	0.577	0.563
CD4 + T cells	0.004	0.002	0.000	0.622	0.538
CD4 + Tcm	0.006	0.004	0.003	2.194	0.115
CD4 + Tem	0.014	0.011	0.011	0.850	0.429
CD8 + naïve T cells	0.008	0.009	0.007	1.943	0.146
CD8 + T cells	0.034	0.015	0.011	10.264	0.000
CD8 + Tcm	0.022	0.014	0.010	3.071	0.049
CD8 + Tem	0.003	0.002	0.000	2.196	0.114
cDC	0.026	0.022	0.019	0.701	0.498
Chondrocytes	0.126	0.161	0.163	4.418	0.013
Class switched memory B cells	0.034	0.025	0.020	1.493	0.228
CLP	0.048	0.042	0.044	1.146	0.320
CMP	0.000	0.000	0.000	1.071	0.345
DC	0.060	0.071	0.056	1.160	0.316
Endothelial cells	0.142	0.143	0.145	0.010	0.990
Eosinophils	0.030	0.036	0.038	3.502	0.032
Epithelial cells	0.726	0.891	0.918	7.364	0.001
Erythrocytes	0.000	0.000	0.000	0.943	0.391
Fibroblasts	0.121	0.138	0.137	0.559	0.573
GMP	0.004	0.002	0.000	2.493	0.086
Hepatocytes	0.085	0.090	0.098	0.516	0.598
HSC	0.196	0.221	0.215	1.009	0.367
iDC	0.119	0.131	0.114	0.591	0.555
Keratinocytes	0.092	0.137	0.142	5.336	0.006
Ly Endothelial cells	0.154	0.157	0.154	0.016	0.984
Macrophages	0.054	0.074	0.072	2.933	0.056
Macrophages M1	0.046	0.065	0.065	5.435	0.005
Macrophages M2	0.015	0.017	0.013	1.055	0.350
Mast cells	0.032	0.029	0.024	3.281	0.040
Megakaryocytes	0.014	0.014	0.012	1.286	0.279
Melanocytes	0.013	0.012	0.011	0.447	0.640
Memory B cells	0.015	0.016	0.009	0.305	0.738
MEP	0.035	0.030	0.027	1.792	0.170
Mesangial cells	0.081	0.093	0.106	8.969	0.000
Monocytes	0.021	0.033	0.021	2.337	0.100
MPP	0.000	0.000	0.000	0.056	0.945
MSC	0.140	0.265	0.310	28.875	0.000
Mv Endothelial cells	0.052	0.057	0.059	0.483	0.618
Myocytes	0.006	0.005	0.004	1.011	0.366
Naïve B cells	0.010	0.013	0.007	0.398	0.672
Neurons	0.066	0.015	0.014	17.406	0.000
Neutrophils	0.001	0.001	0.001	0.094	0.910
NK cells	0.000	0.000	0.000	0.458	0.633
NKT	0.024	0.022	0.026	0.959	0.385
Osteoblast	0.077	0.036	0.034	10.543	0.000
pDC	0.072	0.058	0.046	1.981	0.141
Pericytes	0.074	0.099	0.115	3.779	0.025
Plasma cells	0.011	0.008	0.005	6.987	0.001
Platelets	0.001	0.000	0.000	7.379	0.001
Preadipocytes	0.009	0.002	0.001	3.916	0.022
Pro B cells	0.002	0.002	0.001	0.319	0.727
Sebocytes	0.408	0.533	0.534	4.005	0.020
Skeletal muscle	0.000	0.000	0.000	2.332	0.100
Smooth muscle	0.386	0.332	0.333	12.517	0.000
Tgd cells	0.000	0.000	0.000	0.263	0.769
Th1 cells	0.058	0.046	0.047	1.671	0.191
Th2 cells	0.009	0.010	0.009	0.041	0.960
Tregs	0.016	0.011	0.009	3.210	0.043

*Abbreviations: aDC* Activated dendritic cells, *CD4* + *Tcm*, CD4 + central memory T-cells, *CD4* + *Tem* CD4 + effector memory T-cells, *CD8* + *Tcm* CD8 + central memory T-cells, *CD8* + *Tem* CD8 + effector memory T-cells, *cDC* Xonventional dendritic cells, *CLP* Common lymphoid progenitors, *CMP* Common myeloid progenitors, *DC* Dendritic cells, *GMP* Granulocyte–macrophage progenitors, *HSC* Hematopoietic stem cells, *iDC* Immature dendritic cells, *ly endothelial cells* Lymphatic endothelial cells, *MEP* Megakaryocyte–erythroid progenitors, *MPP* Multipotent rogenitors, *MSC* Mesenchymal stem cells, *mv endothelial cells* Microvascular endothelial cells, *NKT* Natural killer T-cells, *pDC* Plasmacytoid dendritic cells, *Tgd cells* Gamma delta T-cells, *Th1 cells* Type 1 T-helper cells, *Th2 cells* Type 2 T-helper cells, *Tregs* Regulatory T-cells

## Discussion

It has been proved that increased CD8 + T cell infiltration is one of the notable features of immuno-hot tumors, which indicates a better prognosis and high therapeutic response [25–27]. Thus, reliable biomarkers for the identification of immuno-hot tumors in PAAD are urgent in clinical practice. In the current research, we analyzed the expression patterns of B7-H3 and B7-H4 in PAAD and combined their expression as a novel stratification strategy. We found that B7-H3 and B7-H4 were highly expressed in PAAD tissues and higher in poorly differentiated tumors. Moreover, co-deficiency of B7-H3 and B7-H4 indicates a better prognosis and high CD8 + T cell infiltration.

B7-H3 is a negative regulator and inhibits T cell proliferation and cytokine production mediated by antibody to CD3 [28]. In cancers, B7-H3 acts as an inhibitory immune checkpoint that negatively regulates anti-tumor immunity. Overexpression of B7-H3 in tumor tissues is a poor prognostic biomarker in prostate cancer [29], upper tract urothelial carcinoma [30], small cell lung cancer [31], etc. Besides, inhibition of B7-H3 expression is a promising therapeutic strategy for human cancer. In previous research, B7-H3 targeted therapies have been mentioned, which shows promising applications, including monoclonal antibodies against B7-H3, specific antibody-dependent cell-mediated cytotoxicity, antibody drug conjugates, specific small-molecule inhibitor, and chimeric antigen receptor T-cell therapy [21]. B7-H3 expression shows no notable correlation with major TIICs, including CD3 + , CD8 + and CD20 + TIICs in small cell lung cancer [32], whereas B7-H3 expression is positively correlated with the abundance of CD45 + and CD8 + TIICs in non-small-cell lung cancer [33]. In PAAD, B7-H3 was overexpressed and promoted tumor progression [34]. In addition, tumor high B7-H3 expression was independently associated with poor survival [35]. In our research, B7-H3 was positively correlated with CD8 + T cells, CD4 + T cells, neutrophils, macrophages, and DCs estimated by TIMER algorithm, but B7-H3 expression was not correlated with the abundance of CD8 + TIICs in our validated cohort. The contradictory results need to be further confirmed.

Similar to B7-H3, B7-H4 is also an inhibitory immune checkpoint and predicts poor prognosis in multiple human cancers [36–38]. Besides, immunotherapy targeting B7-H4 is under pre-clinical investigation [22]. For example, pharmacologic inhibition of B7-H4 glycosylation restores anti-tumor immunity in immuno-cold breast cancer [39]. It has been reported that B7-H4 expression is inversely correlated with T cell infiltration in clear cell ovarian cancer [40] and breast cancer [24]. In PAAD, B7-H4 promoted cancer progression and inhibited apoptosis in PAAD cells [41]. In addition, a meta-analysis revealed that high expression of B7-H4 was an unfavorable prognostic factor for patients with PAAD [42]. In the current research, we assessed the expression of B7-H4 in tumor and para-tumor tissues in PAAD. However, the results revealed by the GEPIA and CPTAC databases showed no difference between tumor and para-tumor tissues, but IHC staining uncovered that B7-H4 was significantly overexpressed in PAAD tissues. Since B7-H4 was almost only expressed in tumor cells and not in the tumor stroma, we speculated that bulk-RNA sequencing could not distinguish cell subtypes, leading to the false low expression in tumor tissues.

Interestingly, growing numbers of studies have suggested that B7 molecules exhibit limited co-expression patterns [15, 32, 43]. B7-H3 and B7-H4 exhibited no tight correlation in PAAD in our research, but no obvious pattern of mutually exclusive expression was observed as well. Novel prognostic and/or immunogenic classifiers based on different expression patterns of B7 molecules have been preliminarily investigated. For example, B7-H4 is negatively correlated with PD-L1 and identifies immuno-cold tumors in glioma [15]. In our research, we found that co-deficiency of B7-H3 and B7-H4 indicates better prognosis and immuno-hot tumors with high CD8 + T cell infiltration, which could be applied as a novel classifier for prognostic and immunogenic assessment in PAAD.

## Conclusion

To sum up, we analyze the expression patterns and prognostic values of B7-H3 and B7-H4 in PAAD. Single B7-H3 or B7-H4 expression exhibits limited prognostic value for assessment of clinical outcome in PAAD, while combined their expression is a promising stratification strategy to evaluate prognosis and immunogenicity in PAAD.

## Supplementary Information

**Additional file 1:** **Figure S1.** Expression levels of B7-H3 and B7-H4 PAAD tissues based on public data.

## Data Availability

All public data are available in corresponding websites and other necessary data are included in the article. In addition, the current research does not include in-house sequencing data.

## Abbreviations

PAAD: Pancreatic adenocarcinoma
TME: Tumor microenvironment
TIICs: Tumor-infiltrating immune cells
PD-L1: Programmed cell death ligand 1
RNA-seq: RNA-sequencing
TCGA: The Cancer Genome Atlas
TMA: Tissue microarray
IHC: Immunohistochemistry
DAB: Diaminobenzidine
OS: Overall survival
PFS: Progression-free survival
APC: Antigen-presenting cells
