# Supplementary material for: Co-deficiency of B7-H3 and B7-H4 identifies high CD8 + T cell infiltration and better prognosis in pancreatic cancer
Source: BMC Cancer. 2022 Feb 26;22:211. doi: 10.1186/s12885-022-09294-w (PMC8881843; doi:10.1186/s12885-022-09294-w)
Supplement: Supplementary file 1 — Additional file 1: Figure S1. Expression levels of B7-H3 and B7-H4 PAAD tissues based on public data. [file 12885_2022_9294_MOESM1_ESM.docx]

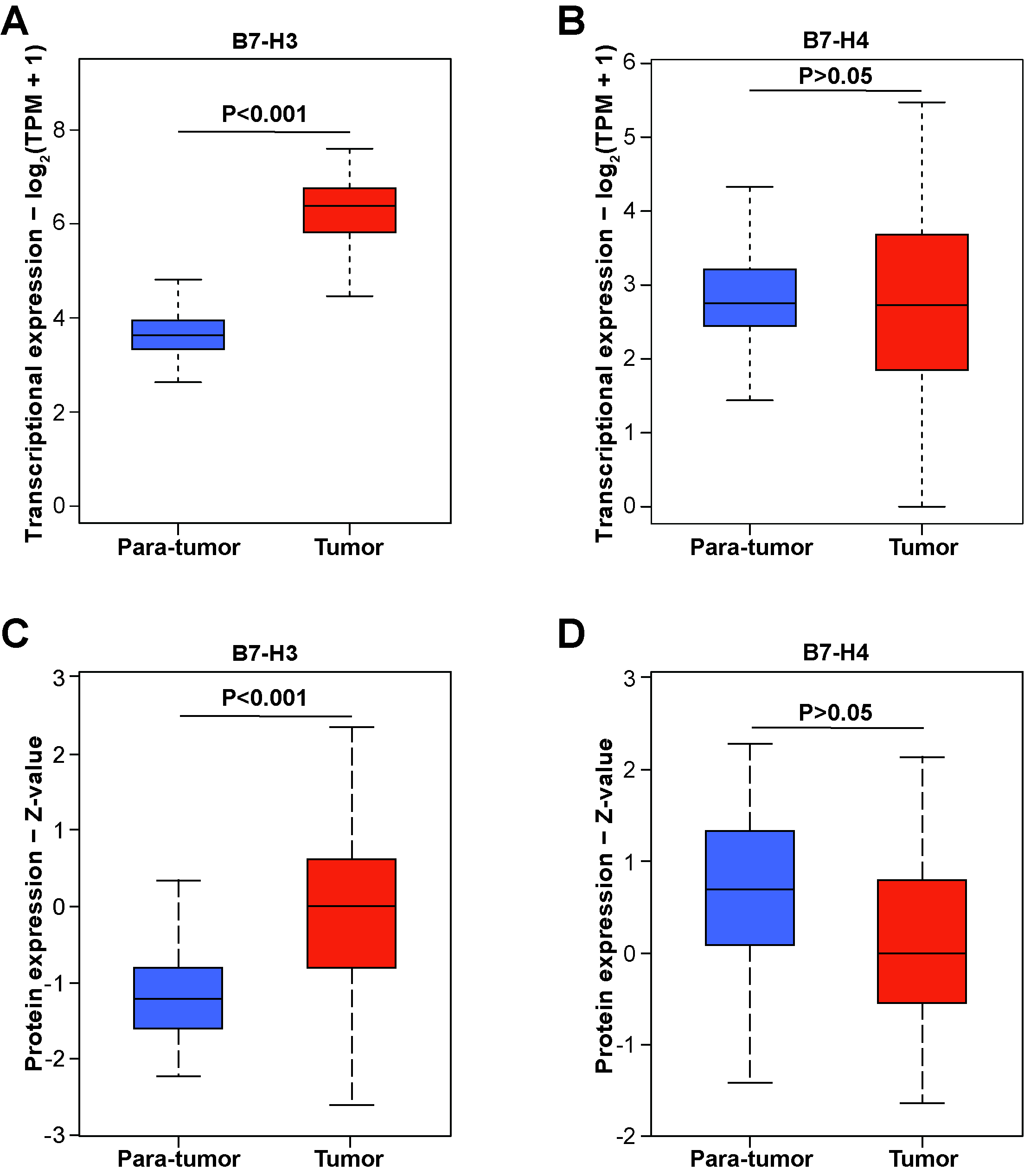


**Figure S1. Expression levels of B7-H3 and B7-H4 PAAD tissues based on public data**

(A, B) B7-H3 was significantly up-regulated, while B7-H4 was no change in tumor tissues compared with para-tumor tissues in the GEPIA database. (C, D) B7-H3 was significantly up-regulated, while B7-H4 was no change in tumor tissues compared with para-tumor tissues in the CPTAC database.
